# Methane Decomposition Over ZrO_2_-Supported Fe and Fe–Ni Catalysts—Effects of Doping La_2_O_3_ and WO_3_

**DOI:** 10.3389/fchem.2020.00317

**Published:** 2020-04-29

**Authors:** Anis H. Fakeeha, Samsudeen Olajide Kasim, Ahmed Aidid Ibrahim, Abdulrhman S. Al-Awadi, Eman Alzahrani, Ahmed Elhag Abasaeed, Ahmed E. Awadallah, Ahmed Sadeq Al-Fatesh

**Affiliations:** ^1^Chemical Engineering Department, College of Engineering, King Saud University, Riyadh, Saudi Arabia; ^2^King Abdullah City for Atomic and Renewable Energy (K.A.CARE), Energy Research and Innovation Center, Riyadh, Saudi Arabia; ^3^Department of Chemistry, Faculty of Science, Taif University, Taif, Saudi Arabia; ^4^Process Development Division, Egyptian Petroleum Research Institute, Cairo, Egypt

**Keywords:** Fe, Fe–Ni, La_2_O_3_ + ZrO_2_, WO_3_ + ZrO_2_, methane conversion, hydrogen, graphitization, Raman spectra

## Abstract

A leading method for hydrogen production that is free of carbon oxides is catalytic methane decomposition. In this research, Fe and Fe–Ni supported catalysts prepared by the wet impregnation method were used in methane decomposition. The effects of doping the parent support (ZrO_2_) with La_2_O_3_ and WO_3_ were studied. It was discovered that the support doped with La_2_O_3_ gave the best performance in terms of CH_4_ conversion, H_2_ yield, and stability at the test condition, 800°C and 4,000-ml h^−1^ g^−1^ cat. space velocity. The addition of Ni significantly improved the performance of all the monometallic catalysts. The catalysts were characterized by X-ray diffraction (XRD), Brunauer–Emmett–Teller (BET), temperature-programmed reduction/oxidation (TPR/TPO), thermogravimetric analyzer (TGA), and microscopy (SEM and Raman) techniques. Phases of the different forms of Fe were identified by XRD. BET showed a drastic decline in the specific surface area of the catalysts with respect to the supports. TPR profiles revealed a progressive change in the valency of Fe in its combined form to the zero valence-free metal. The La_2_O_3_-promoted support gave the best performance and maintained good stability during the time on stream.

## Introduction

Methane (CH_4_) is the main component of natural and biogas. Its use as a feed is expected to increase in the current year due to greenhouse gas effects (Fakeeha et al., 2015; Calgaro and Perez-Lopez, 2019). Global warming has been a great concern for mankind. Emissions of greenhouse gases like CH_4_ contribute aggressively to environmental issues. Methods of transforming CH_4_ into handy products are worthy from the prospect of safety and the economic point of view of generating value-added fuels and chemicals (Ashok et al., 2008; Muhammad et al., 2018; Zhang et al., 2018). In this context, a direct approach is chosen for hydrogen (H_2_) and elemental carbon production, as given in Equation (1). H_2_, which has been considered to be among the greenest and lightest fuel, is pivotal in the broad requirement of energy, while the carbon that is produced as a by-product could function as a value-added product in power generation and as a catalyst. A filamentous form of carbon and graphene, which is formed from the catalytic methane decomposition (CMD), is highly important in nanoscience because of its distinctive properties (i.e., electrical, chemical, and mechanical) (Pudukudy et al., 2016). Another form of carbon that is of interest and that can be obtained using this process is graphene (Jana et al., 2011; Ibrahim et al., 2015; Pudukudy et al., 2016). It has gained much attention in light of its excellent properties, such as its extraordinary chemical stability, large surface area, and good structural strength and conductivity (electrical and thermal) (Li et al., 2011; Wang and Lau, 2015; Ashik et al., 2017).

Furthermore, other methods by which H_2_ could be generated from CH_4_ exist. These include steam reforming, partial oxidation, and hydrogen sulfide methane reformation. Steam reforming is the cheapest source of H_2_ at the moment, and it involves heating methane to around 700–1,100°C in the presence of steam and a catalyst (e.g., nickel). However, the downside of this process is that CO, CO_2_, and other greenhouse gases are its major by-products. A ton of H_2_ produced will consequently have 9–12 ton of CO_2_ being produced alongside (Collodi, 2010).

Equation (1) implies a reaction that takes place at higher temperatures; consequently, the applications of catalysts are in binding to reduce the activation energy (Ashik et al., 2017). To enhance the CH_4_ decomposition reaction, metal-based catalysts were often used by researchers. These include transition metals such as Fe and Ni with a partially filled d-orbital. These metals are characterized by a low price and better stability and activity (Ashik et al., 2015; Inaba et al., 2019). Supported Ni-based catalysts are preferable for the CMD process due to their high activity, cheap price, and wide availability.

(1) $$\begin{array}{l} \text{C}{\text{H}}_{4}\;\to \text{ C }+\;2{\text{H}}_{2}\;74.9\text{ kJ mo}{\text{l}}^{-1} \end{array}$$

Karaismailoglu et al. investigated catalytic methane decomposition using yttria-doped nickel-based catalysts (Karaismailoglu et al., 2019). They prepared their catalyst using a sol–gel technique and studied the activity in the temperature range between 390° and 845°C. Their results showed that increasing the temperature favored the formation of coke, and the CH_4_ conversions of 14 and 50% at 500° and 800°C, respectively, were attained.

Another study that is of interest is methane decomposition without catalyst pre-reduction. This was aimed at reducing the operating cost of the whole process, and thus, reduction of the catalysts in the gas feed stream was performed. However, the hydrogen yields for such processes were reported to be low (Enakonda et al., 2016; Musamali and Isa, 2018).

Transition metals (e.g., Ni, Co, and Fe) supported on different oxides have been the widely used catalysts in CMD reactions (Cunha et al., 2009; Shen and Lua, 2015). The effects of different supports such as La_2_O_3_, ZrO_2_, SiO_2_, Al_2_O_3_, SiO_2_/Al_2_O_3_, and TiO_2_ on Ni-based catalysts have been studied (Muraza and Galadima, 2015; Khan et al., 2016). Furthermore, promising results were obtained in the investigation of Fe over La_2_O_3_ at the temperature range of 500–750°C (Ibrahim et al., 2018). As at the time of preparing this report, no study has been reported in the literature on catalytic methane decomposition using Fe supported on zirconia (ZrO_2_) and WO_3_. More so, the properties of a single metal catalyst have been reported to be enhanced *via* the introduction of a second metal, therefore leading to a bimetallic catalyst idea (Pudukudy et al., 2014). This study will also investigate the effect of adding Ni to the Fe supported catalysts.

The major problem of the CMD is the rapid deactivation of the catalyst caused by the deposition of amorphous carbon on the surface of the catalyst. This low-activity carbon covers the active metal particle (Calgaro and Perez-Lopez, 2017). It has been established that Ni catalysts are effective for methane decomposition reaction at temperatures of 500–600°C, which are below the equilibrium-required temperature. At higher temperatures, Ni catalysts deactivate rapidly, but Fe can withstand these necessary high temperatures. Also, Fe is relatively cheaper than Ni (Inaba et al., 2002).

During this investigation, monometallic Fe- and bimetallic Fe/Ni-based catalysts supported over zirconia and modified zirconia were used for the catalytic decomposition of CH_4_. The effects of the catalyst composition, in terms of active metals and the support modification, were evaluated based on the characteristics of the catalyst's stability and activity. The best catalyst composition has been determined. The efficiency of the catalysts in the decomposition of CH_4_, with respect to activity and stability, was studied.

## Materials and Methods

### Catalyst Preparation

Monometallic supported Fe catalysts, as well as the bimetallic supported catalysts (Fe and Ni) used in this study, were synthesized using the technique called wet impregnation. The supports (ZrO_2_, 9%La_2_O_3_-ZrO_2_, and 10%WO_3_-ZrO_2_) were obtained from Daiichi Kigenso Kagaku Kogyo Co., Ltd. (Osaka, Japan), and the authors are really grateful for the support. Hydrated iron nitrate [Fe(NO_3_)_3_·9H_2_O, 99%] was used as the active metal for the monometallic supported catalysts, while iron and nickel nitrate salts were combined in appropriate proportions and used as the active metals in the bimetallic supported catalysts. The percentage loading of Fe was 40 wt% for the monometallic and 20 wt% for each one of Fe and Ni in the bimetallic supported catalysts. The stoichiometric amounts of all active metals were measured and added to distilled water (30 ml), followed by the supports. The support-active metal mixtures, present in separate crucibles, were stirred and dried at 80°C for 3 h over different hot plates. Subsequently, the samples were placed inside an oven for overnight drying at 120°C. Calcination of the samples was done at 800°C for 3 h in the oven.

### Catalyst Activity

The CMD study was performed using Fe and bimetallic Fe–Ni supported catalysts in an upright, fixed-bed, stainless steel tubular micro-reactor (PID Eng&Tech microactivity reference), 9.1-mm ID and 30 cm long, at atmospheric pressure. Catalyst testing was performed using a catalyst mass of 0.3 g, which was carefully positioned over a bed of glass wool inside the reactor. The actual reactor temperature was read by an axially positioned thermocouple (K-type), sheathed in stainless steel. The total time of analysis for each of the catalysts was 240 min. Before the start of the reaction, each of the catalysts was reduced under the flow of H_2_ at 20 ml/min for 90 min at 800°C. Thereafter, the system was purged with N_2_ for 15 min to remove any remnant of H_2_. The temperature of the reactor was raised to that required for the reaction (i.e., 800°C) in the flow of N_2_. The feed gas mixture was maintained at a total flow rate of 20 ml/min (13 and 7 ml/min for CH_4_ and N_2_, respectively) and an equivalent space velocity of 4,000 m h^−1^ g^−1^-cat. The product gas composition was analyzed by gas chromatography (GC; Shimadzu, 2004), which was connected online. The GC is equipped with a thermal conductivity detector (TCD). The following expressions were used to determine the methane conversion and hydrogen yield:

$$\begin{array}{l} \text{ C}{\text{H}}_{4}\text{ conversion }=\;\frac{\text{C}{\text{H}}_{4}\text{ in }-\text{ C}{\text{H}}_{4}\text{ out}}{\text{C}{\text{H}}_{4}\text{ in}}\;\times \;100\text{\%}\\ {\text{H}}_{2}\text{ Yield}:\;{\text{Y}}_{{\text{H}}_{2}}\;=\;\frac{\text{moles of }{\text{H}}_{2}\text{ produced}}{2\;\times \text{ moles of C}{\text{H}}_{4}\text{ in the feed}}\times 100\text{\%} \end{array}$$

### Catalyst Characterization

The phase formation and crystal structure of fresh catalyst samples were examined using an X-ray diffractometer (XRD). A Miniflex Rigaku diffractometer, having CuKα X-ray radiation, that operates at 40 kV and 40 mA was employed for the examination. The XRD pattern was taken at a diffraction angle (2θ) between 10 and 80° and a step size of 0.01°. X'pert HighScore Plus software was used to analyze the raw data file of the instrument. Different phases with their scores were matched with the Joint Committee of Powder Diffraction Standards (JCPDS) data bank.

The Micromeritics TriStar II 3020 surface area and porosity analyzer was used in the determination of the textural characteristics of the fresh samples. The analysis was done by N_2_ physisorption performed at −196°C. Prior to the analysis, each sample was degassed at 200°C for 3 h in the flow of nitrogen gas. The specific surface area and pore volume of the catalyst samples were calculated by the Brunauer–Emmett–Teller (BET) and Barret–Joyner–Halenda (BJH) methods, respectively.

A Thermo Scientific X-ray photoelectron spectrometer (XPS) was used in recording the XPS data of the fresh catalyst samples. High-resolution scan was achieved using a monochromated Al Kα (1,486.6 eV) radiation source running at a power of 72 W with a pass energy of 50 eV–200 eV for survey scans.

Temperature-programmed reduction/oxidation (TPR/TPO) was performed on the fresh and spent catalysts, respectively, using Micromeritics AutoChem II 2920. For the TPR measurement, 70 mg of the catalyst samples were placed inside the sample tube and then carefully positioned in the machine's furnace. Thereafter, the sample was pretreated by flushing with argon at 150°C for an hour and then cooled to 23°C. Eventually, the furnace temperature was raised to 1,000°C at the ramp of 10°C/min in the presence of a H_2_/Ar mixture flowing at 40 ml/min. A cold trap within the machine removes the water produced during the reduction process, while a thermal conductivity detector records the H_2_ that was being consumed. For the TPO, measurements were carried out in the presence of oxygen to ascertain the kind of carbon that was deposited onto the surface of the used catalysts. Spent catalysts were subjected to the same pretreatment as in TPR, and the analysis was done at a range of 50–900°C in a mixture of 10% O_2_/He flowing at 40 ml/min.

A scanning electron microscope was used to study the changes in the morphology of the calcined samples. A JEOL JSM-7100F (JEOL, Tokyo, Japan) field-emission scanning electron microscope was used for this study.

The determination of the amount of carbon deposits was carried out using a Shimadzu thermogravimetric analyzer (TGA). The temperature of the spent catalysts (10–15 mg) was raised from 23°C up to 1,000°C at the rate of 20°C/min, and the mass difference was recorded by the machine.

Raman spectra were obtained using an NMR-4500 laser Raman spectrometer (JASCO, Japan). The excitation beam was configured to a wavelength of 532 nm. The measurement was done using an objective lens with × 20 magnification. The beam power was set to 6 mW and the exposure time to 3 min to protect the sample from being damaged by laser irradiation. The spectra were measured in the range 500–3,000 cm^−1^ (Raman shift), while Spectra Manager Ver.2 software (JASCO, Japan) was used to process them.

## Results and Discussion

### X-Ray Diffraction

**Figure 1:**
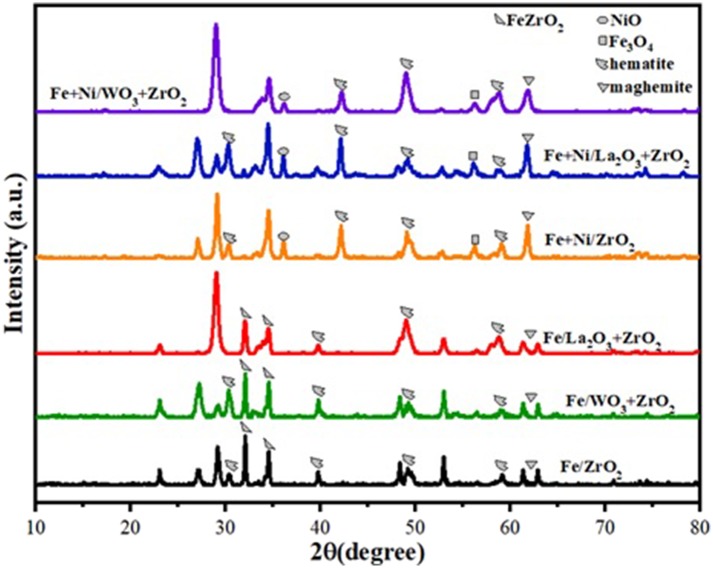
XRD diffractograms for the Fe and Fe–Ni supported catalysts.

The XRD diffractograms of the fresh Fe and Fe–Ni supported samples are presented in Figure 1. The XRD revealed the different phases existing on the catalysts. Monometallic catalysts exhibit almost the same peaks at the same 2θ angle.

On the one hand, hematite (Fe_2_O_3_) (JCPDS card no. 33-0664 at 2θ angles of 30, 40, 50, and 59°) and maghemite Fe_2_O_3_ (JCPDS card no. 00-039-1346 at 2θ angles of 35 and 62°) phases were obtained in both Fe and Fe–Ni supported catalysts. On the other hand, magnetite (Fe_3_O_4_, JCPDS card no. 00-019-0629 at 2θ angle of 56°) was obtained in the XRD diffractogram of the Fe–Ni supported catalysts. Besides, a NiO phase was noticed on the bimetallic catalysts at 2θ angle of 36°. The remaining diffraction peaks could be assigned to the tetragonal and monoclinic zirconia phases. It can also be seen that the relative intensities of all the diffraction peaks of Fe supported on the La_2_O_3_ + ZrO_2_ catalyst were more pronounced compared with those of the other catalysts. This indicates that the introduction of La_2_O_3_ in the catalyst structure improved the dispersion of the metal particles.

### Surface Characterization

**Figure 2:**
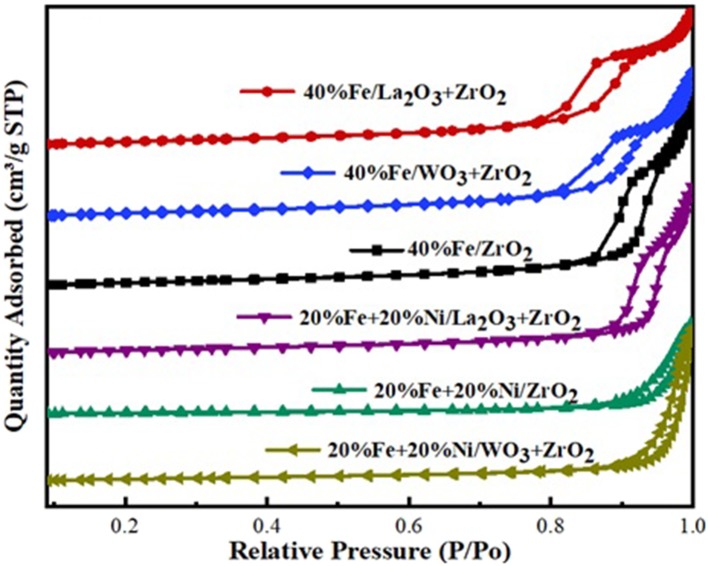
Adsorption–desorption isotherms.

**Figure 3:**
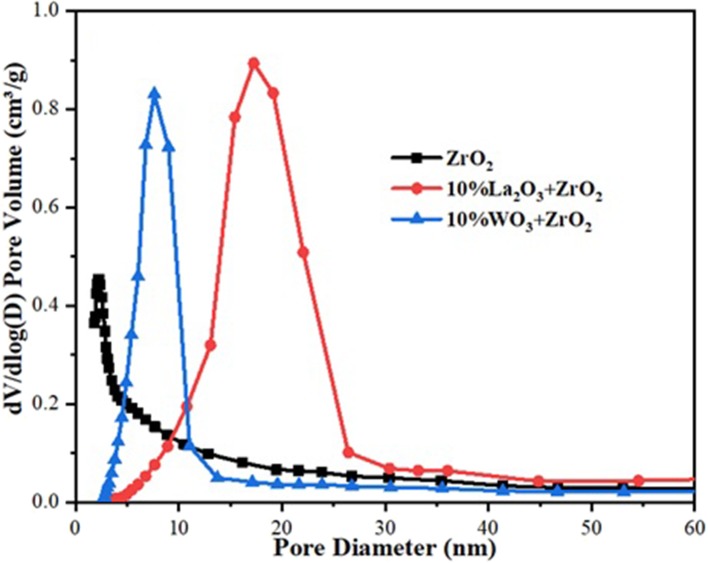
Pore size distribution of Fe and Fe–Ni supported catalysts.

The BET analysis showed the textural properties of the different supports and catalysts. Tables 1A,B contain the results of the analysis for the supports as well as that of the synthesized catalysts, respectively. Also, the N_2_ adsorption–desorption isotherms are shown in Figure 2. An active metal addition to the support consequently led to a drastic decrease in the supports' surface area, probably due to the blockage of the pores of the supports. These isotherms belong to the type IV category, according to the IUPAC classification. Also, all isotherms are characterized by capillary condensation at a higher relative pressure, which is typical of mesoporous materials. The pore size distribution of the supports is shown in Figure 3. From the figure, the pore diameters of the samples are within the range of 2–50 nm. It was observed that the addition of Ni led to an increase in the surface area of the catalysts, except for 20%Fe + 20%Ni/ZrO_2_. This could be due to the doping effect and the aggregation of the metal particles due to the weak metal–support interaction. The explanation and figure on surface atom identification can be found in Supplementary File.

**Table 1A:** N_2_ physisorption results for the supports.

**Catalyst**	**Specific surface area (m^**2**^/g)**	**Particle size D50 (μm)**
ZrO_2_	325	29.7
10%WO_3_+ZrO_2_	112	3.70
9%La_2_O_3_+ZrO_2_	67.3	4.04

**Table 1B:** N_2_ physisorption results for the synthesized catalysts.

**Catalyst**	**BET surface** **area (m**^**2**^**/g)**	**Av. Pore** **diameter (nm)**	**Pore volume** **(cm**^**3**^**/g)**
40%Fe/ZrO_2_	11.23	36.88	0.09
40%Fe/La_2_O_3_+ZrO_2_	16.34	30.14	0.11
40%Fe/WO_3_+ZrO_2_	21.75	20.33	0.10
20%Fe+20%Ni/ZrO_2_	7.16	43.55	0.06
20%Fe+20%Ni/ La_2_O_3_+ZrO_2_	21.12	27.37	0.13
20%Fe+20%Ni/ WO_3_+ZrO_2_	23.36	18.19	0.01

### H_2_ Temperature-Programmed Reduction

**Figure 4:**
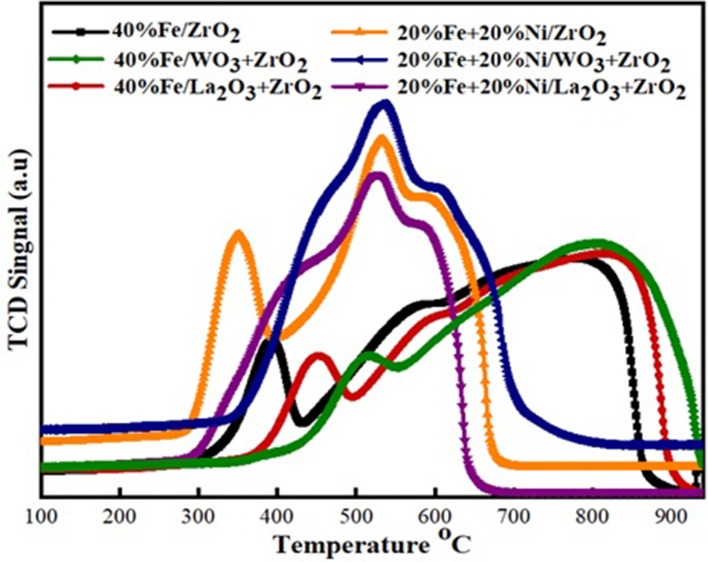
Temperature-programmed reduction (TPR) profiles of the single metal and bimetallic supported catalysts.

H_2_ temperature-programmed reduction (H_2_-TPR) was performed to investigate the reducibility and the extent of the metal–support interaction of the calcined single metal (40 wt%Fe)-based catalysts as well as the bimetallic (20 wt% Fe and 20 wt% Ni) counterpart. As shown in Figure 4, the H_2_-TPR profiles for the single metal-supported catalysts (i.e., 40%Fe/ZrO_2_, 40%Fe/La_2_O_3_ + ZrO_2_, and 40%Fe/WO_3_ + ZrO_2_) showed three distinct reduction peaks at different temperature ranges. The peaks show the progressive reduction of Fe_2_O_3_ to zero valence Fe (Fe_2_O_3_ → Fe_3_O_4_ → FeO → Fe). The reduction peak within 300–500°C could be attributed to the reduction of Fe_2_O_3_ to FeO·Fe_2_O_3_, while the peaks that appeared within the temperature range of 500–700°C could be assigned to the further reduction of Fe_2_O_3_ from Fe_3_O_4_ to FeO. Finally, the temperature range of 700–850°C showed the reduction peaks for FeO to metallic Fe particles. A similar reduction behavior was studied by Bayat et al. while investigating the decomposition of methane over Ni–Fe/Al_2_O_3_ catalysts intended for the production of CO_*x*_-free hydrogen and carbon nanofiber (Bayat et al., 2016). On the one hand, the peaks for 40%Fe/WO_3_ + ZrO_2_ appeared at higher temperature ranges relative to the other single metal-supported catalysts. This could be attributed to the existence of a stronger interaction between Fe and the support. On the other hand, the reduction peaks for 40%Fe/La_2_O_3_ + ZrO_2_ seemed to be at the intermediate relative to the other single metal-supported catalysts, i.e., the Fe metal is neither weakly nor strongly bound to the support to the extent that will make its activation difficult. H_2_ consumption corresponding to the reduction peaks that were obtained during the H_2_-TPR analysis is shown in Table 2. From this table, 40%Fe/La_2_O_3_ + ZrO_2_ has the least H_2_ uptake from among the single metal-supported catalysts, which implies that it could be activated with ease at moderate temperatures.

**Table 2:** Quantitative analysis of H_2_ consumption during H_2_-TPR.

**Samples**	**Temperature** **(**^**°**^**C)**	**Quantity consumed** **(μmol/g)**	**Total**
40%Fe/ZrO_2_	363	692.53	5117.28
	628	4192.77	
	743	231.98	
40%Fe/La_2_O_3_+ZrO_2_	383	213.13	4531.15
	594	4184.96	
	754	126.86	
	981	6.20	
40%Fe/WO_3_+ZrO_2_	496	150.96	5487.09
	578	30.42	
	744	5305.71	
20%Fe+20%Ni/ZrO_2_	351	1786.02	8037.23
	533	6251.21	
20%Fe+20%Ni/ La_2_O_3_+ZrO_2_	528	7328.56	7328.56
20%Fe+20%Ni/ WO_3_+ZrO_2_	533	7713.91	7713.91

The same trend of reduction was observed for the bimetallic catalyst samples. The observed difference is that peaks of higher intensity/H_2_ consumption were observed for the bimetallic samples owing to the presence of an additional metal oxide, i.e., NiO. Moreover, the reduction peak of NiO at a temperature of ~350°C for the Fe–Ni/ZrO_2_ catalyst disappeared after incorporating La_2_O_3_ and WO_3_ in the catalyst compositions. This further demonstrates the role of these dopants in enhancing the metal dispersion. From Table 2, the average H_2_ consumptions for the mono- and bimetallic samples are 5,045 and 7,693 μmol/g, respectively. Also, the reduction peaks for the bimetallic samples appeared at lower temperatures relative to the single metal-supported samples. This suggests that the addition of NiO does not only influence the reduction behavior of the catalysts but also improve their reducibility.

### Scanning Electron Microscopy

**Figure 5:**
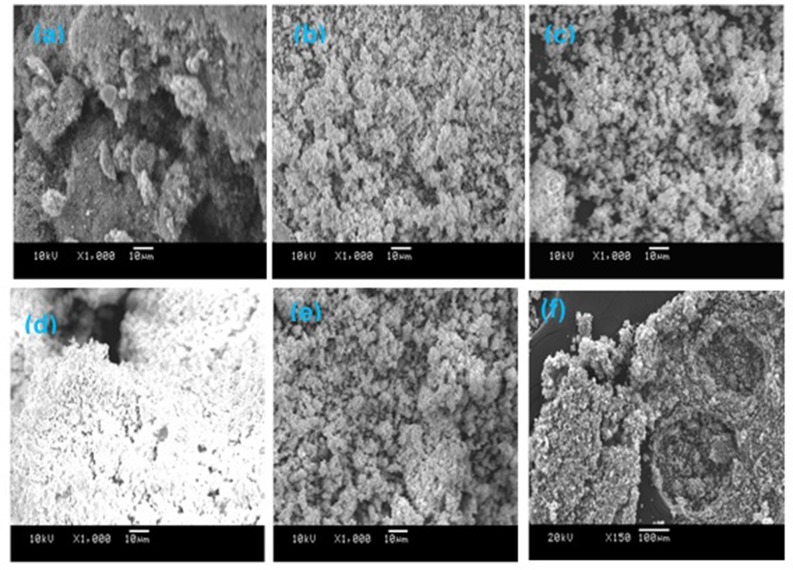
SEM images of fresh calcined catalysts. **(a)** 40%Fe/ZrO2; **(b)** 40%Fe/La_2_O_3_ + ZrO_2_; **(c)** 40%Fe/WO_3_ + ZrO_2_; **(d)** 20%Fe + 20%Ni/ZrO_2_; **(e)** 20%Fe + 20%Ni/WO_3_ + ZrO_2_; and **(f)** spent 40%Fe/ZrO_2_.

The morphologies of the freshly calcined monometallic Fe- and bimetallic Fe/Ni-containing catalysts were investigated using the SEM technique. The images are displayed in Figure 5. The SEM images were recorded at the same magnification to study the change in the surface morphology of the catalysts depending on the type of dopants. As illustrated in Figure 4A, the particles of the Fe/ZrO_2_ catalyst were found to be large clusters with unclear crystal interfaces and irregular sizes. This manifests that the Fe oxide particles were randomly aggregated on the surface of the zirconia support. Upon the addition of La_2_O_3_ and WO_3_ to the Fe/ZrO_2_ catalyst, the particle sizes became smaller, more separated, and better distributed (Figures 5B,C). A similar surface morphology was also observed for the bimetallic Fe/Ni-containing catalysts, indicating that the addition of Ni also improved the dispersion of themetal particles (Figures 5D,E). The fresh samples have a homogeneous morphology compared to the spent sample (Figure 5F). The heterogeneous nature of the morphology of the spent sample is suggestive of the presence of an additional substance (e.g., carbon) that was formed during the reaction.

### Catalyst Activity

**Figure 6:**
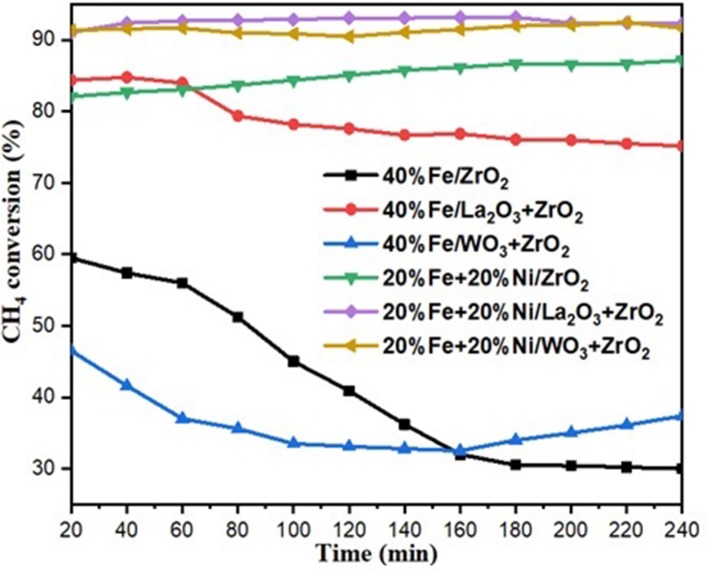
CH_4_ conversion for the catalysts under investigation.

**Figure 7:**
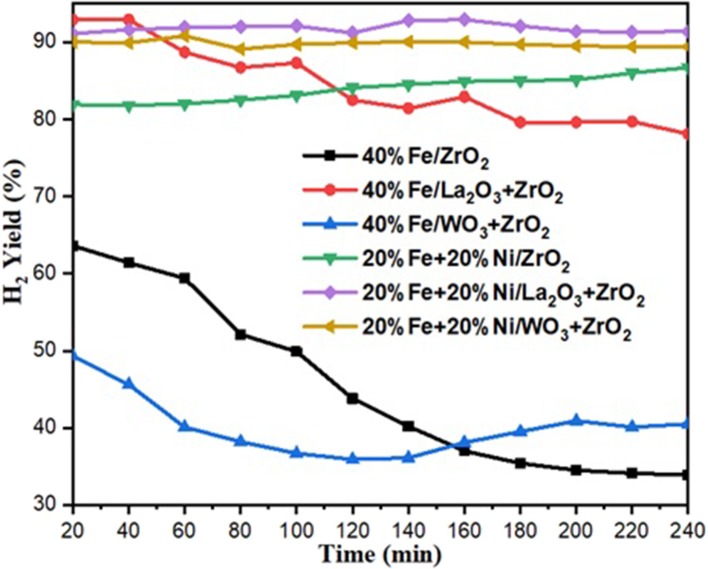
H_2_ yield for the catalysts under investigation.

The catalytic methane decomposition results over 240-min time on stream using 40 wt% Fe and 20 wt% Fe–Ni supported catalysts are shown in Figures 6, 7. The feed was maintained at 4,000-ml h^−1^ g^−1^ cat. space velocity and the reaction was performed at an atmospheric pressure and temperature of 800°C. The results show the effect of doping the primary support (ZrO_2_) for the single metal-supported catalysts as well as the effect of adding Ni. From the figure, 40%Fe/La_2_O_3_ + ZrO_2_ gave the highest CH_4_ conversion of about 79% compared with 40%Fe/ZrO_2_ and 40%Fe/La_2_O_3_ + ZrO_2_, both having 42 and 36%, respectively. 40%Fe/ZrO_2_ and 40%Fe/WO_3_ + ZrO_2_ began with a considerable CH_4_ conversion of about 60 and 46%, respectively, but suffered a fast deactivation throughout the study. Fe supported on WO_3_ + ZrO_2_, i.e., 40%Fe/WO_3_ + ZrO_2_, had the least conversion, while the catalyst having its support doped with La_2_O_3_ (i.e., 40%Fe/La_2_O_3_ + ZrO_2_) did not only give a higher performance but also showed the best stability for the period of reaction. The poor performance of the Fe/ZrO_2_ may not be unconnected to its low specific surface area. And for Fe/WO_3_ + ZrO_2_, it appeared that the addition of WO_3_ increased the metal–support interaction to the detriment of the availability of the Fe for the reaction.

For the bimetallic catalysts, the addition of 20 wt% Ni improved the CH_4_ conversion as well as the stability. The conversion of CH_4_ increased to about 85, 91, and 92% for Fe–Ni/ZrO_2_, Fe–Ni/WO_3_ + ZrO_2_, and Fe–Ni/La_2_O_3_ + ZrO_2_, respectively.

Anis et al. reported a similar behavior, where the addition of Ni enhanced the Fe/Al_2_O_3_ catalyst's performance in their study of the effect of activation temperature, in their paper “*Bimetallic Catalysts of Mesoporous Al*_2_*O*_3_ *Supported on Fe, Ni and Mn for Methane Decomposition: Effect of Activation Temperature*.” It was discovered that adding Ni, in an equal amount of Fe, to Fe/Al_2_O_3_ led to a relatively higher CH_4_ conversion of 61% during the reaction (Fakeeha et al., 2018).

Hydrogen yield for the monometallic catalysts revealed that 40%Fe/WO_3_ + ZrO_2_ gave the least H_2_ yield (about 40%), followed by 40%Fe/ZrO_2_ (45%), while the 40%Fe/La_2_O_3_ + ZrO_2_ catalyst had the highest H_2_ yield of about 84%. The best performance of the Fe catalyst supported on lanthanum-doped ZrO_2_ is expected, as the BET results showed that, relatively, it has a considerable specific surface area and the largest pore volume^1^. Ahmed et al. has reported in their findings that higher surface area and pore volume usually enhance catalyst activity (Ahmed et al., 2015). Moreover, the improved metal dispersion after incorporating La_2_O_3_ into the Fe/ZrO_2_ catalyst could also be responsible for its higher activity and stability. The addition of Ni to Fe/ZrO_2_ and Fe/WO_3_ + ZrO_2_ as an active metal greatly raised the H_2_ yield to about 84 and 90%, respectively. Also, the catalysts were observed to be stable throughout the investigation. A similar improvement was observed for the Fe–Ni/La_2_O_3_ + ZrO_2_ catalyst, as the H_2_ yield increased from 84%, for its monometallic, to 92%. These results are presented in Figures 6, 7. From the results, it can be inferred that, among all the dopants used with ZrO_2_, La_2_O_3_ improved the catalyst's activity.

Table 3 shows the comparison of the catalytic activities reported in the literature, on methane decomposition, with that of the present work. Some catalysts showed high initial conversion and lost their activities while the reaction was going on. Thus, it suffices to say that 20%Fe + 20%Ni/La_2_O_3_ + ZrO_2_ demonstrated the best conversion and stability over the other catalysts being compared.

**Table 3:** Comparison of catalytic performance with published results.

**Catalysts**	**Temperature (^**°**^C)**	**GHSV^**a**^ (mL/(gh))**	**CH_**4**_ conversion (%)**	**References**
40%Fe/Al_2_O_3_	750	6,000	75	Qian et al., 2019
Ni-Fe/Al_2_O_3_	800	75,000	60	Tezel et al., 2019
2.5Ni-Y/SiO_2_	800	60,000	9	Karaismailoglu et al., 2019
55Ni/MgO	600	48,000	65	Rastegarpanah et al., 2019
40%Fe/La_2_O_3_+ZrO_2_	800	4,000	79	Present work
20%Fe+Ni/La_2_O_3_+ZrO_2_	800	4,000	92	Present work

a *GHSV, Gas Hourly Space Velocity*.

### Thermal Analysis (TGA)

**Figure 8:**
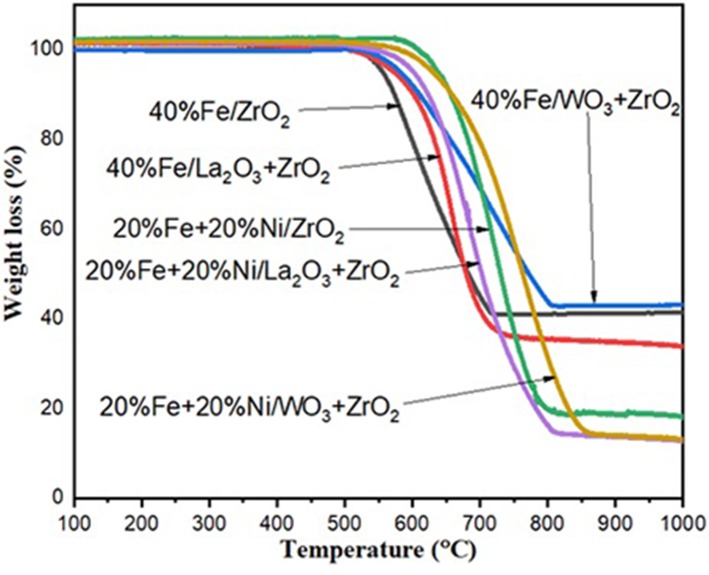
Thermogravimetric analysis (TGA) results for Fe/ZrO_2_, Fe/La_2_O_3_ + ZrO_2_, and Fe/WO_3_ + ZrO_2_ and their corresponding bimetallic catalysts.

After 240 min of reaction, the spent catalysts were collected from the reactor and subjected to TGA to determine the amount of carbon deposited during the reaction. Figure 8 shows the TGA curves that translate to the quantitative amount of carbon deposits for all the used catalysts. The total carbon deposited on the spent catalysts was expressed as a weight percentage. For the set of monometallic catalysts, Fe/La_2_O_3_ + ZrO_2_ has the highest amount of carbon deposit of about 65%, while Fe/WO_3_ + ZrO_2_ has the least carbon deposit equivalent to 57% weight loss. This is in agreement with the results obtained from their CH_4_ conversion.

For the bimetallic catalysts, a similar trend was noticed as the addition of Ni increased the activity of the catalysts, with the most active catalyst having the highest amount of carbon deposits.

From these results, it can be inferred that the addition of Ni enhanced the conversion of CH_4_ in all cases and consequently led to a higher carbon deposit since CH_4_ is the only source of carbon.

### Temperature-Programmed Oxidation

TPO is an essential characterization method useful in determining the kind of deposited carbon found on spent catalysts. The different kinds of carbon formed on a catalyst's surface during the CMD could be removed at different temperature ranges: <250°C (atomic carbon), 250–600°C (amorphous carbon), and >600°C (graphitic carbon) (Hao et al., 2009). In general, the carbon in the shape of a filament has been reported to be formed on the metal catalysts used in methane decomposition reaction (Takenaka et al., 2004; Chen et al., 2005; Nuernberg et al., 2008).

**Figure 9:**
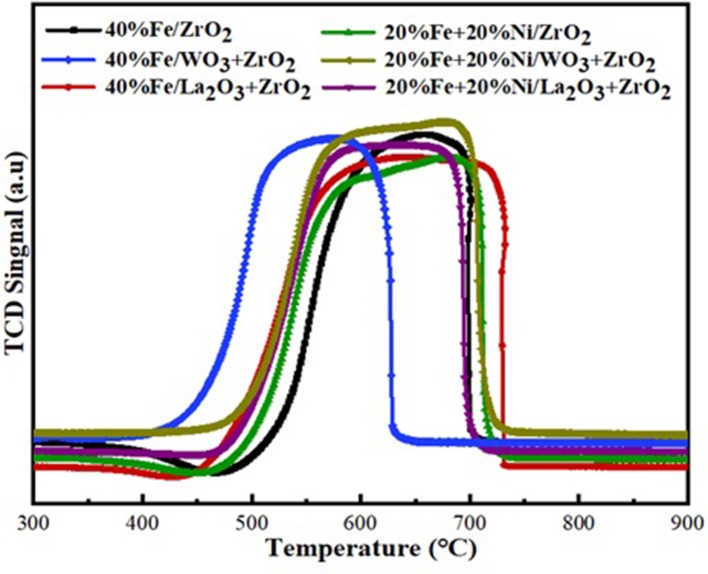
Temperature-programmed oxidation (TPO) profiles for the spent catalysts.

From the TPO results in Figure 9, the carbon deposits on Fe/WO_3_ + ZrO_2_ lie within the temperature range of amorphous and partly graphitic carbon. The deposits on Fe/ZrO_2_ and Fe/La_2_O_3_ + ZrO_2_ spanned across the amorphous and graphitic range.

Similarly, for the used bimetallic catalysts, the TPO curves appeared in the range of amorphous and graphitic carbon. All the bimetallic catalysts were observed to have higher activity and stability, as seen in the activity figure; owing to that, the addition of Ni promoted the formation of filamentous–graphitic carbon whose growth does not hinder access to the active metal sites. Zhang et al. reported in their study that the formation and growth of filamentous carbons during catalytic methane decomposition was beneficial in keeping the active Ni sites accessible for CH_4_ molecules and, consequently, made it possible for the catalysts to maintain their activity and stability for a longer time (Zhanga et al., 2019).

### Raman Spectroscopic Analysis

**Figure 10:**
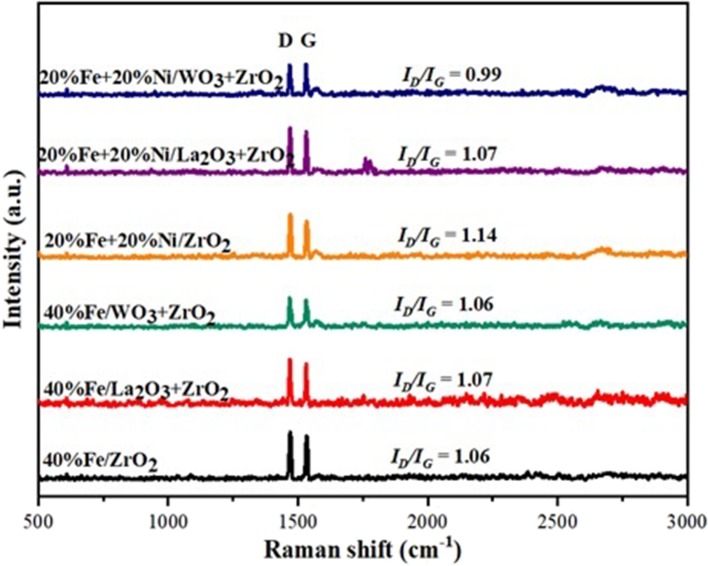
Raman spectra for the used samples.

Raman spectroscopic analysis was performed to study the structure of carbon deposits over the spent catalysts. The obtained Raman spectra are shown in Figure 10 below. For all the spent samples, two main spectra were observed at ~1,470 and 1,532 cm^−1^, corresponding to the D and G bands, respectively. The G band can be attributed to the ideal vibration of the graphite layers as a result of the in-plane carbon–carbon stretching. The D band (disorder mode) is attributed to the structural defect of graphite (Dresselhaus et al., 2010). Besides, a small shoulder (D′) that appeared at 1,596 cm^−1^ can be related to the disordered carbons at the edge (Darmstadt et al., 1997). The 2D band at 2,718 cm^−1^ can be regarded as an overtone of the D band at 1,470 cm^−1^, but it does not depend on the density of defect. In general, the ratio of the intensity of the D band to the G band (i.e., *I*_D_*/I*_G_) gives a measure of the crystalline order of graphite in carbonaceous materials (Kameya and Hanamura, 2011). This implies that the degree of graphitization is higher for small values of *I*_D_*/I*_G_ and *vice versa*. The *I*_D_*/I*_G_ of all the spent catalysts shown in Figure 8 indicate that the carbon deposits over the catalysts have almost equal disordered amorphous and graphitic structures.

### XRD of the Reduced Catalysts

**Figure 11:**
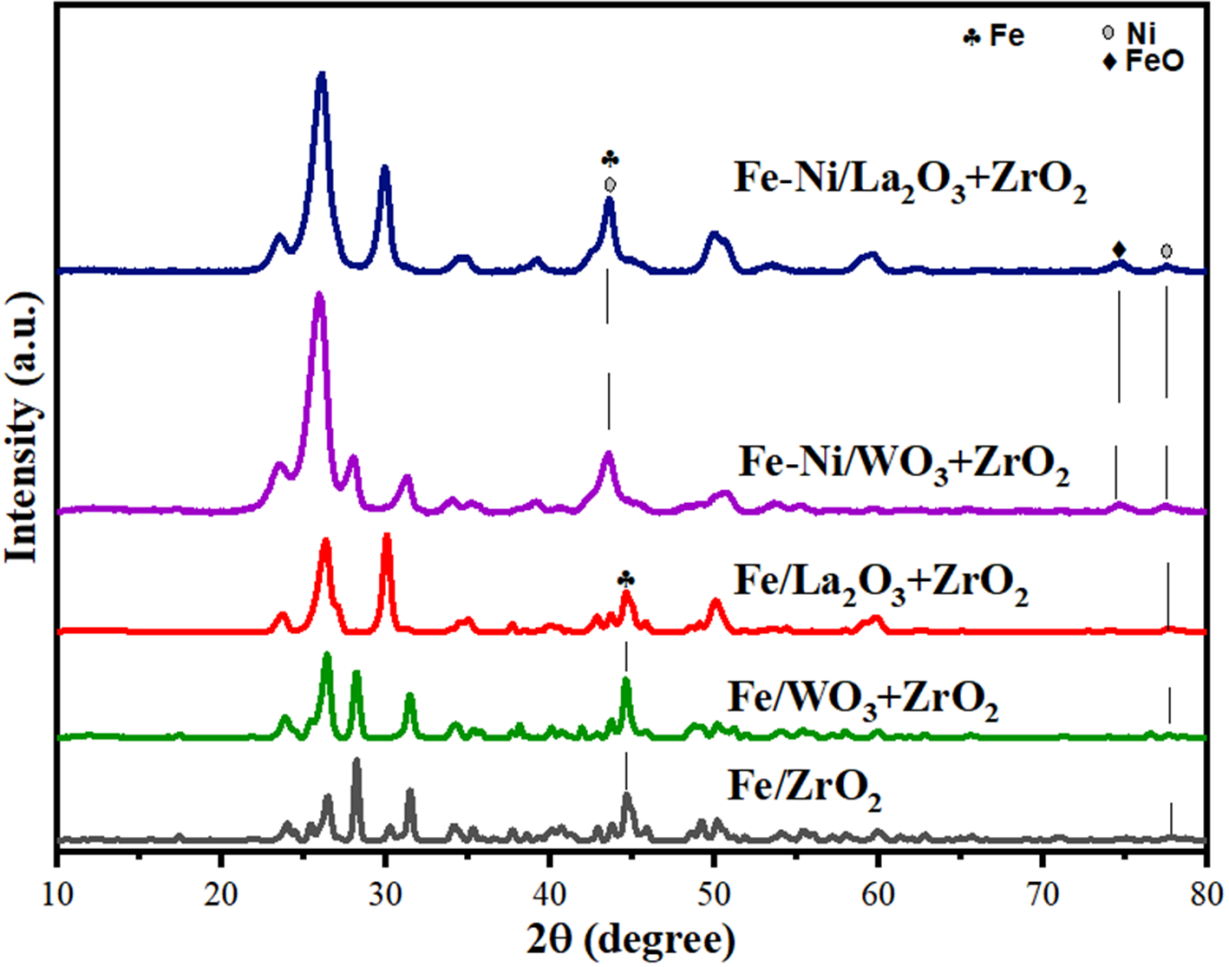
XRD diffractograms of the reduced catalysts.

XRD analysis was performed on the reduced catalysts to investigate the state of the catalysts after being reduced under the flow of H_2_. The Fe in the fresh samples exists majorly in the form of Fe_2_O_3_ (hematite), and a substantial reduction in the peaks of Fe_2_O_3_ can be seen in the diffractograms of the reduced samples, indicating the reduction of the hematite. The peaks corresponding to the reduced FeO, Fe, and Ni have been identified in Figure 11 concerning the analysis done by Ren et al. (2015). All other peaks are as identified in the fresh catalyst samples.

## Conclusion

In this study, the wet impregnation method was used in the synthesis of Fe and Fe–Ni supported catalysts. The synthesized catalysts were used in catalytic methane decomposition. ZrO_2_ was the primary support and was doped with metal oxides such as La_2_O_3_ and WO_3_. This paper investigates the effect of doping the primary support (ZrO_2_) as well as the effect of adding Ni. From the activity of the catalysts, Fe/La_2_O_3_ + ZrO_2_ gave the highest methane conversion as well as hydrogen yield (79 and 84%, respectively) among the monometallic catalysts. This catalyst is seen to be the most stable. It was observed that the addition of Ni improved not only the performance of the catalysts but also their stability. In all cases, Ni enhanced the performance of the catalysts.

The fresh and used catalysts were subjected to many characterization techniques. The synthesized catalysts' surface area decreased significantly concerning the corresponding support. TPR showed the progressive reduction of iron(III) oxide to the zero valence free metal. The same trend was observed for both Fe and Fe–Ni supported catalysts. Also, a higher peak intensity was noticed for the bimetallic supported catalysts. The thermogravimetric analysis revealed a high amount of carbon deposit for both Fe and Fe–Ni supported on La_2_O_3_ + ZrO_2_. The temperature-programmed oxidation showed that amorphous and graphitic carbons were the kinds of carbon deposited over the spent catalysts for the time on stream studied. The carbon deposits over the spent catalysts were characterized by a mixture of amorphous and graphitic carbon that are filamentous.

## Data Availability Statement

The raw data supporting the conclusions of this article will be made available by the authors, without undue reservation, to any qualified researcher.

## Author Contributions

AA-F, SK, and AI synthesized the catalysts, carried out all the experiments and characterization tests, and wrote the manuscript. AA-A and EA analysis XRD, SEM. AAb, AAw, and AF writing-review and editing.

## Conflict of Interest

The authors declare that the research was conducted in the absence of any commercial or financial relationships that could be construed as a potential conflict of interest.

## Footnotes

^1^https://xpssimplified.com/elements/lanthanum.php
