# Supplementary material for: Methane Decomposition Over ZrO2-Supported Fe and Fe–Ni Catalysts—Effects of Doping La2O3 and WO3
Source: Front Chem. 2020 Apr 29;8:317. doi: 10.3389/fchem.2020.00317 (PMC7201101; doi:10.3389/fchem.2020.00317)
Supplement: Supplementary file 1 [file Data_Sheet_1.docx]

**X-ray photoelectron spectroscopy (XPS)**

The chemical composition of the surface of the fresh catalyst was determined using X-ray photoelectron spectroscopy (XPS). The La(4p), Zr(3d), O(1s), Fe(2p), and Ni(2p) XPS spectra of Fe-Ni/La_2_O_3_+ZrO_2_ are shown in figure S1.

The Zr(3d) XPS spectra seen at electron voltage values of 177 and 180 can be attributed to the photoelectrons from the Zr(3d5/2). The La(4p3/2) spectra could be seen appearing at 185 and 188 eV. These values are below the reference value reported for pure La_2_O_3_ (Thermoscientific, 2020). This shift in energy value could be due to the incorporation of other metals. From figure S1c, the binding energy values of the O(1s) at 523 and 532 eV indicate an overlapping contribution of oxygen from the support (La_2_O_3_+ZrO_2_) as reported by Reddy (Reddy et al., 2001). The Fe(2p) XPS spectra show peaks that are typical of magnetite with both Fe^2+^ and Fe^3+^contributing their own quota. From figure S1d, electrons that were ejected from Fe(2p3/2) are the dominant. Furthermore, the Ni(2p) XPS spectra of the sample showed peaks that are typical of Ni^2+^(2p3/2) at binding energy values that are equivalent to 852 and 855 eV. This was also reported by Ertl, in their study on XPS of cupper aluminate (Ertl et al., 1980).


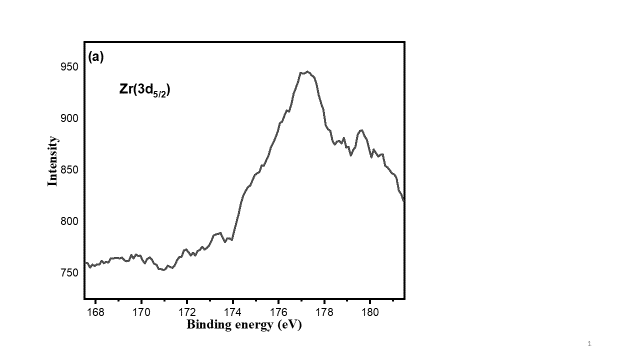

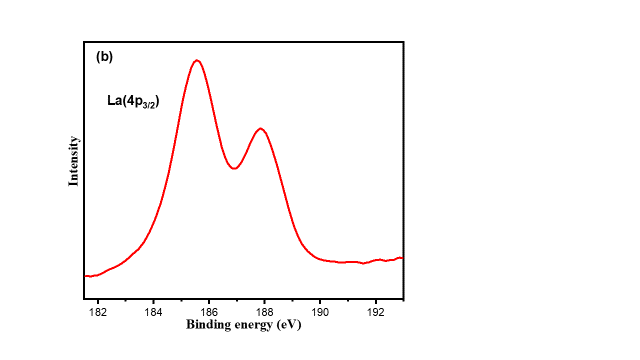


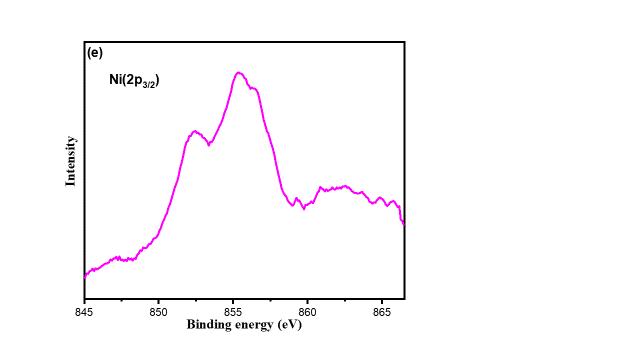

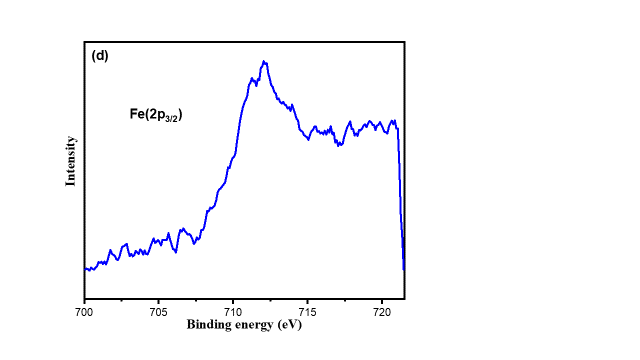

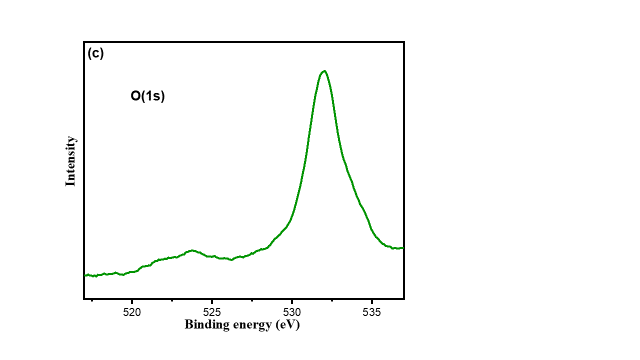


Figure S: XPS spectra of fresh 20%Fe+Ni/La_2_O_3_+ZrO_2_ sample.

REFERENCES

Ertl, G., Hierl, R., Knozinger, H., Thiele, N., and Urbach, H. P. (1980). XPS study of copper aluminate catalysts. *Appl. Surf. Sci.* 5, 49–64. doi: 10.1016/0378-5963(80)90117-8

Reddy, B. M., Chowdhury, B., and Smirniotis, P. G. (2001). An XPS study of the dispersion of MoO3 on TiO2-ZrO2, TiO2-SiO2, TiO2-Al2O3, SiO2- ZrO2, and SiO2-TiO2-ZrO2 mixed oxides. *Appl. Catal. A Gen*. 211, 19–30. doi: 10.1016/S0926-860X(00)00834-6
